# Diagnostic accuracy of contrast-enhanced computed tomography in assessing cervical lymph node status in patients with oral squamous cell carcinoma

**DOI:** 10.1007/s00432-023-05470-y

**Published:** 2023-10-25

**Authors:** Ann-Kristin Struckmeier, Ebrahim Yekta, Abbas Agaimy, Markus Kopp, Mayte Buchbender, Tobias Moest, Rainer Lutz, Marco Kesting

**Affiliations:** 1 Department of Oral and Cranio-Maxillofacial Surgery, Friedrich-Alexander-Universität Erlangen-Nürnberg (FAU), Glückstraße 11, 91054 Erlangen, Germany; 2 Comprehensive Cancer Center Erlangen-European Metropolitan Area of Nuremberg (CCC ER-EMN), Erlangen, Germany; 3 Institute of Pathology, Friedrich-Alexander-Universität Erlangen-Nürnberg (FAU), Erlangen, Germany; 4 Department of Radiology, Friedrich-Alexander-Universität Erlangen-Nürnberg (FAU), Erlangen, Germany

**Keywords:** Computed tomography, Lymph node metastasis, Diagnostic accuracy, Oral squamous cell carcinoma

## Abstract

**Objective:**

Accurate preoperative prediction of lymph node (LN) status plays a pivotal role in determining the extension of neck dissection (ND) required for patients with oral squamous cell carcinoma (OSCC). This study aims to evaluate the diagnostic accuracy of contrast-enhanced computed tomography (CT) in detecting LN metastases (LNMs) and to explore clinicopathological factors associated with its reliability.

**Methods:**

Data from 239 patients with primary OSCC who underwent preoperative CT and subsequent radical surgery involving ND were retrospectively reviewed. Suspicious LNs were categorized into three groups: accentuated (< 10 mm), enlarged (≥ 10 mm), and melted. Statistical analysis encompassing correlation and comparative analysis, and determination of sensitivity, specificity, PPV, and NPV were performed.

**Results:**

Overall, sensitivity was significantly higher in the accentuated LNs group (83.54%) compared to the melted LNs group (39.24%, *p* < 0.05, *t* test). Conversely, specificity was significantly higher in the melted LNs group (98.19%) compared to the accentuated LNs group (55.15%, *p* < 0.05, *t* test). Accentuated LNs exhibited a false negative rate of 13.00%. False positive rates were 51.80%, 30.26% and 8.82%, respectively. Diagnostic accuracy for detecting LNMs in level IIa and IIb exceeded that of level III. Patients with solely accentuated LNs were more likely to have a small, well-differentiated tumor. However, no distinctions emerged in terms of the occurrence of T4 tumors among the three groups.

**Conclusion:**

CT proves sufficient to predict LNMs in patients with OSCC. Looking ahead, the potential integration of artificial intelligence and deep learning holds promise to further enhance the reliability of CT in LNMs detection. However, this prospect necessitates further investigation.

**Supplementary Information:**

The online version contains supplementary material available at 10.1007/s00432-023-05470-y.

## Introduction

Oral squamous cell carcinoma (OSCC) constitutes approximately 90% of all malignant neoplasms of the oral cavity and has a global annual incidence exceeding 350,000 cases (Ferlay et al. 2019; Massano et al. 2006).

The gold standard of treatment involves the surgical resection accomplished by a (microvascular) defect closure following oncology board meetings’ recommendation. As OSCC is characterized by a high propensity for cervical lymph node metastases (LNMs; 42.6%) (Moratin et al. 2020), the recommended approach also includes concurrent neck dissection (ND) (Crile 1906). The metastatic dissemination patterns generally hinge on the primary tumor’s location and have been studied in previous series (Moratin et al. 2020; Woolgar 2007).

According to the German guideline for OSCC therapy, a contrast-enhanced computed tomography (CT) or magnetic resonance imaging (MRI) should be performed preoperatively to determine the local extent of the tumor and to identify potential LNMs (DGMKG 2021). However, currently, there are no standard criteria in imaging to definitively designate lymph nodes (LNs) as metastatic in OSCC patients. Nonetheless, distinct radiological characteristics may indicate a suspicious LN. Non-metastatic LNs typically appear as discrete, kidney-shaped structures composed of soft-tissue, featuring a concave hilum consisting of fat tissue. In contrast, LNs with metastases appear round in imaging and exhibit a rim enhancement, along with irregular borders, a non-uniform parenchymal staining pattern, and central attenuation (Som 1992). Additionally, size remains a commonly used criterion, typically ranging from 5 to 15 mm (Curtin et al. 1998). Nevertheless, the precise assessment of cervical LNMs through imaging remains an unresolved issue.

LN status significantly influences treatment decisions for oncology patients. Broadly, a distinction is made between elective and therapeutic NDs. An elective ND is carried out in case of no clinical evidence of LNMs, whereas a therapeutic ND is undertaken if clinical or imaging evidence of LNMs is present at pretreatment diagnosis of OSCC.

**Fig. 1:**
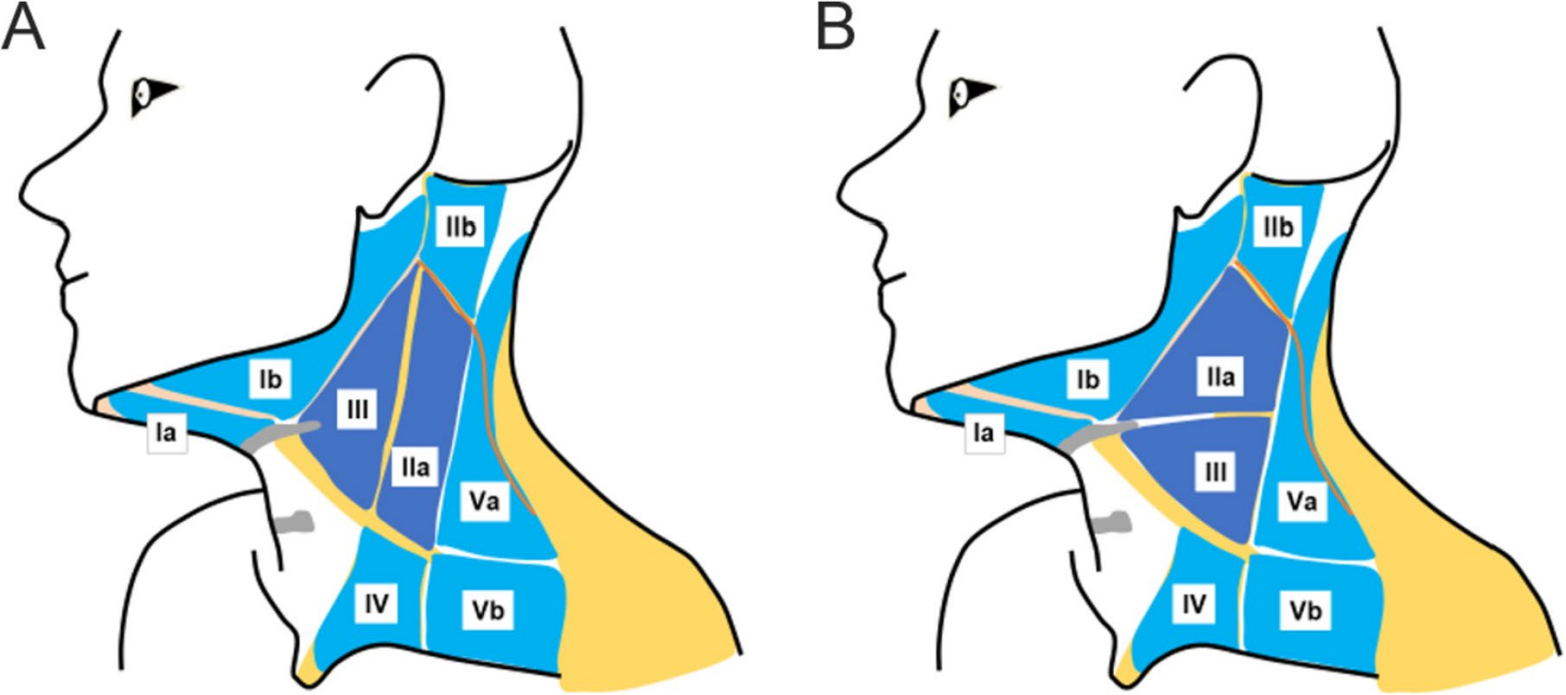
Graphic schemes of the neck’s topographical areas as categorized by **A** Kesting and **B** Robbins. Kesting’s classification places emphasis on surgically important landmarks (e.g., the internal jugular vein), whereas Robbins’ classification relies on anatomical landmarks (e.g., level of the hyoid bone). The two classifications diverge in their definitions of level IIa and level III. According to Kesting’s classification, Level IIa is situated below the sternocleidomastoid muscle, posterior to the internal jugular vein, anterior the posterior border of the sternocleidomastoid muscle, cranial to the crossing point of the omohyoid muscle and sternocleidomastoid muscle, and caudal to the spinal accessory nerve. Additionally, Kesting defines level III as a triangle formed by the internal jugular vein, the omohyoid muscle, and the posterior belly of the digastric muscle. On the contrary, Robbins defines level II as the lymph nodes located posterior to the stylohyoid muscle and anterior the posterior border of the sternocleidomastoid muscle. Lymph nodes from level IIa are located caudal to the spinal accessory nerve and cranial to the level of the inferior border of the hyoid bone. Robbins delineates Level III as a triangle defined by the lateral border of the sternohyoid muscle, the posterior border of the sternocleidomastoid muscle, and the inferior border of the hyoid bone

The neck’s topographical areas are categorized into levels based on anatomical landmarks, as outlined by Robbins et al. (2002, 1991) or in modification by Kesting (2015) and Koerdt et al. (2016). Kesting introduced a classification system and a sequential algorithm for neck dissection that emphasizes surgical technical aspects over anatomical topography, diverging from the approach advocated by Robbins (Kesting 2015). Figure 1 illustrates both classifications.

In cases of clinically node negative (cN0) neck, a SND, i.e., supraomohyoid ND including the levels I–III, is typically performed ipsilateral, due to an approximately 25% risk of occult LNMs (D’Cruz et al. 2015; Fasunla et al. 2011). For tumors that are midline or approaching the midline, a bilateral SND is recommended (Florke et al. 2021). In case of intraoperative (i.e., by frozen section technique) or postoperative evidence of ipsilateral LNM, the SND is extended to an ipsilateral MRND along with a contralateral SND (Koerdt et al. 2016). In the presence of contralateral LNM, a strong suggestion is made for extending the contralateral SND to a MRND (Lim et al. 2006).

This approach captures the majority of LNMs while minimizing associated morbidity (Teymoortash et al. 2010). However, this approach carries the risk of potentially missing LNMs that may not align with the usual lymphatic drainage patterns (Byers et al. 1997).

Historically, ND were conducted en bloc (Upile et al. 2007). Nowadays, split up ND are preferred, since splitting the lymph node specimens into lymph node packages provides information on the precise location of LNMs after histopathological examination (Kesting 2015; Koerdt et al. 2016). This enables decisions about the necessity for extending the neck dissection to level IV and V and tailoring adjuvant radiation therapy (Koerdt et al. 2016). On the contrary, there is no established clinical evidence supporting the superiority of en bloc resections.

Overall, the LN status holds significant implications in determining the extent of ND required for patients with OSCC. Consequently, the precise anticipation of LN status preoperatively, i.e., before pathological confirmation, plays a pivotal role for clinicians. This underscores the importance of selecting an appropriate imaging method to ensure a precise assessment of LN status.

However, the current body of knowledge presents a range of diverse and varied data concerning the reliability of CT imaging in accurately identifying LNMs in OSCC patients preoperatively.

The primary objective of this study was to assess the precision of contrast-enhanced CT in detecting cervical LNMs in OSCC patients by comparing preoperative CT imaging results with subsequent histopathological findings. Furthermore, we conducted comparative analyses to delve deeper into the impact of different clinicopathological characteristics on the diagnostic accuracy of CT.

## Materials and methods

### Study design and participants

A retrospective investigation was conducted within a cohort comprising 239 patients diagnosed with primary OSCC. All patients underwent staging with contrast-enhanced CT and primary surgical treatment including tumor resection and ND followed by histopathological examination of all tissue specimens at the Department of Oral and Maxillofacial Surgery at the University Hospital Erlangen. ND in each patient comprised at least the ipsilateral supraomohyoid levels, i.e., the levels I–III.

The treatment regimen adhered to the national OSCC therapy guideline. Time point of diagnosis spanned from October 1, 2017, to October 31, 2022. Patients with recurrent OSCC and those who did not undergo ND or ND with a decreased extent due to severe comorbidities were excluded. The inclusion and exclusion criteria are presented in Table S1.

Various parameters were meticulously documented and subjected to assessment, encompassing age, sex, tumor localization, pathological TNM classification, histological grading, histopathological evidence of perineural invasion, lymphovascular invasion, vascular invasion, extranodal extension, and the histopathologically confirmed levels with LNMs. All characteristics were assembled from hospital medical records and the tumors were classified according to the 8th UICC edition.

During surgery, the lymph node specimens were resected as part of a split up ND. Subsequently, the surgeon labeled the lymph node packages using the previously mentioned modified version of the Robbins classification by Kesting (2015) and Koerdt et al. (2016).

The tissue samples were subsequently sent to the Department of Pathology for histopathological analysis. In case of macroscopic impression of LNM intraoperatively, LNs were promptly examined using the frozen section technique. The Department of Pathology at the University Hospital Erlangen provided the histopathological diagnosis and tumor differentiation grade.

In accordance with national regulations and institutional regulations, written informed consent was not required from the participating patients.

The Ethics Committee of the Friedrich-Alexander University Erlangen-Nuremberg approved the study’s design and methods (Ethic votes: 23-185-Br, 23-186-Br).

Our study adhered to the Standards of Reporting of Diagnostic Accuracy (STARD) reporting guideline for diagnostic studies.

### Contrast-enhanced computed tomography

Thin-section axial multidetector CT scans were performed for all patients enrolled in this study, utilizing a minimal slice thickness of 1 mm. Additionally, sagittal and coronal multiplanar reconstructions (slice thickness of 3 mm) were generated. The CT scanners utilized were SOMATOM Definition AS + and SOMATOM X.ceed from Siemens Healthineers (Erlangen, Germany).

All CT scans were performed with an injection of intravenous iodine-based contrast agent to enhance differentiation of soft tissues (Imeron 350 mg/mL, Bracco Group, Milan, Italy; flow rate of 3 mL/s).

The evaluation of CT datasets involved a minimum of two independent physicians from the Department of Radiology. At least one consultant assessed the local extent of the tumor and evaluated LN status.

**Fig. 2:**
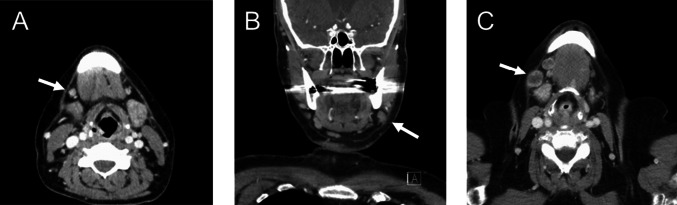
Representative examples of **A** accentuated, **B** enlarged, and **C** melted lymph nodes in level Ib

Suspicious LNs in contrast-enhanced CT were categorized based on their appearance as accelerated (< 10 mm), enlarged (≥ 10 mm), and melted. For assessing the size of LNs, the short axial diameter was measured. Lymph nodes were categorized as accentuated if they displayed enlargement, particularly in side-by-side comparison, while still maintaining a size under 10 mm. We defined necrosis in melted lymph nodes as a central area with low attenuation surrounded by an irregular rim of enhancing tissue. Representative CT images illustrating various LN appearances are provided in Fig. 2.

### Statistical analysis

Statistical analysis was performed using the Statistical Package for the Social Sciences 27.0 (SPSS, Chicago, IL, USA). All graphs were created with Excel 2016 (Microsoft, Redmond, WA, USA).

Correlation analysis was performed using chi-square and spearman’s rank correlation test depending on the variables. Student’s t test was used in order to compare the means between groups.

Generally, a *p* value < 0.05 was considered statistically significant.

The diagnostic accuracy of contrast-enhanced CT imaging was determined by calculating sensitivity, specificity, positive predictive value (PPV), and negative predictive value (NPV). Histopathological results were used as gold standard.

## Results

### Patient cohort

The study encompassed a cohort of 239 individuals with primary OSCC. Among these, 155 (64.85%) were male, and 84 (35.15%) were female. The age range spanned from 31 to 88 years, with a median age of 65 years. Tumor localization was predominantly observed at the floor of the mouth (*n* = 65; 27.20%), lateral tongue (*n* = 63; 26.36%), and lower jaw (*n* = 48; 20.08%).

Oncologic therapy included an ipsilateral SND in 127 (53.14%) patients, bilateral SND in 61 (25.52%) patients, ipsilateral MRND combined with contralateral SND in 40 (16.73%) patients, and bilateral MRND in 8 (3.35%) patients. The number of resected LNs ranged between 9 and 121, with a median of 36. Simultaneously, the number of resected metastatic LNs varied between 0 and 23.

The clinicopathological characteristics of the patient cohort are summarized in Table 1.

**Table 1:** Clinicopathological characteristics of the investigated cohort

Characteristics	Number of patients (%)
No. of patients	239
Sex	
Male	155 (64.85)
Female	84 (35.15)
Age	
Median	65
Range	31–88
Tumor localization	
Floor of the mouth	65 (27.20)
Lateral tongue	63 (26.36)
Lower jaw	48 (20.08)
Upper jaw	29 (12.13)
Buccal plane	17 (7.11)
Palate	16 (6.69)
Multilocular	1 (0.42)
Type of neck dissection	
Ipsilateral SND	127 (53.14)
Ipsilateral MRND	3 (1.26)
Bilateral SND	61 (25.52)
Ipsilateral MRND + contralateral SND	40 (16.73)
Bilateral MRND	8 (3.35)
No. of resected LNs	
Median	36
Range	9–121
No. of resected metastatic LNs	
Median	0
Range	0–23
Histological grading	
G1	28 (11.72)
G2	118 (49.37)
G3	89 (37.24)
Gx	4 (1.67)
Pathological T stage	
T1	90 (37.67)
T2	59 (24.69)
T3	33 (13.81)
T4	55 (23.01)
Pathological N stage	
N0	159 (66.53)
N1	31 (12.97)
N2a	5 (2.09)
N2b	18 (7.53)
N2c	5 (2.09)
N3b	21 (8.79)
Extranodal extension (% of LNMs)	
ENE (−)	52 (65.00)
ENE (+)	28 (35.00)
Lymphovascular invasion	
L0	224 (93.72)
L1	15 (6.28)
Vascular invasion	
V0	236 (98.74)
V1	3 (1.26)
Perineural invasion	
Pn0	202 (84.52)
Pn1	37 (15.48)

*LN* lymph node, *LNM* lymph node metastasis, *MRND* modified radical neck dissection, *SND* selective neck dissection

### Reliability of computed tomography depending on cervical level

Histopathological analysis unveiled LNMs in various levels according to modified Robbins’ classification by Kesting: level Ia (*n* = 6), level Ib (*n* = 44), level IIa (*n* = 26), level IIb (*n* = 14), level III (*n* = 27), level IV (n = 3), and level V (*n* = 2). Notably, LNM presentation was most reliable in CT in level IIa and IIb, where 88.46% and 92.86% of LNMs were accentuated, 76.92% and 78.57% were enlarged, and 53.85% and 57.14% were melted. Conversely, in level III, only 62.96% of LNMs were enlarged in CT, and merely 44.44% were melted. The limited occurrence of LNMs in level Ia and levels IV and V precluded meaningful assessment. Results based on the localization of the LNMs and their appearance in the preoperative CT scans are outlined in Table 2.

**Table 2:** Cervical levels harboring lymph node metastasis confirmed in histopathological examination categorized based on their appearance in contrast-enhanced computed tomography

Characteristics of the LNs	Lymph node levels of the neck						
	Ia (%)	Ib (%)	IIa (%)	IIb (%)	III (%)	IV (%)	V (%)
Accentuated LN	6 (100)	38 (86.36)	23 (88.46)	13 (92.86)	23 (85.19)	3 (100)	2 (100)
Inconspicuous LN	0 (0)	6 (13.64)	3 (11.54)	1 (7.14)	4 (14.81)	0 (0)	0 (0)
Enlarged LN positive	6 (100)	32 (72.73)	20 (76.92)	11 (78.57)	17 (62.96)	3 (100)	2 (100)
Enlarged LN negative	0 (0)	12 (27.27)	6 (23.08)	3 (21.43)	10 (37.04)	0 (0)	0 (0)
Melted LN positive	3 (50)	20 (45.45)	14 (53.85)	8 (57.14)	12 (44.44)	2 (66.67)	1 (50)
Melted LN negative	3 (50)	24 (54.54)	12 (46.15)	6 (42.86)	15 (55.56)	1 (33.33)	1 (50)
Overall	6	44	26	14	27	3	2

Suspicious lymph nodes in computed tomography were categorized as accelerated (< 10 mm), enlarged (≥ 10 mm), and melted

*LN* lymph node

### Correlation of the appearance of lymph nodes in computed tomography with clinicopathological characteristics

A correlation analysis was then performed between the appearance of LNs on CT and clinicopathological characteristics. First, the clinicopathological characteristics of patients without suspicious LNs on CT were examined.

Patients staged as cN0, mostly had tumors localized at the floor of the mouth and underwent ipsilateral SND in 60% of the cases, while bilateral SND was performed in 32% of the patients. As expected, the number of resected LNs (median 32) and the number of LNMs (median 0) was lower in patients with cN0 neck compared to the patients with suspicious LNs. cN0 status was confirmed in 87% of the patients. Notably, 13% of the patients had LNMs despite the absence of suspicious LNs in preoperative CT (false negative rate).

Detailed clinicopathological characteristics based on LN appearance in CT scan and results of correlation analysis are presented in Tables 3 and 4.

**Table 3:** Correlation of inconspicuous, accentuated (< 10 mm), enlarged (≥ 10 mm), and melted lymph nodes in contrast-enhanced computer tomography with clinical characteristics of oral squamous cell carcinoma patients

Characteristics	Inconspicuous (%)	*p* value	Accentuated (%)	*p* value	Enlarged (%)	*p* value	Melted (%)	*p* value
No. of patients	100 (41.84)		139 (58.16)		76 (31.38)		34 (24.46)	
Sex		0.158		0.170		0.658		0.735
Male	70 (70)		85 (61.15)		48 (63.16)		21 (61.76)	
Female	30 (30)		54 (38.85)		28 (36.84)		13 (38.24)	
Age		0.385		0.278		0.146		0.432
Median	65		65		64		64	
Range	33–88		31–88		38–87		38–87	
Tumor localization		0.067		0.048*		0.511		0.137
Floor of the mouth	32 (32)		33 (23.74)		20 (26.32)		12 (35.29)	
Lateral tongue	26 (26)		37 (26.62)		17 (22.37)		6 (17.65)	
Lower jaw	15 (15)		33 (23.74)		15 (19.74)		7 (20.59)	
Upper jaw	8 (8)		21 (15.11)		13 (17.11)		5 (14.71)	
Buccal plane	8 (8)		9 (6.47)		5 (6.58)		1 (2.94)	
Palate	11 (11)		5 (3.60)		5 (6.58)		2 (5.88)	
Multilocular	0 (0)		1 (0.72)		1 (1.32)		1 (2.94)	
Type of neck dissection		< 0.001*		< 0.001*		< 0.001*		< 0.001*
Ipsilateral SND	60 (60)		67 (48.20)		27 (35.53)		6 (17.65)	
Ipsilateral MRND	0 (0)		3 (2.16)		3 (3.95)		1 (2.94)	
Bilateral SND	34 (34)		27 (19.42)		13 (17.11)		6 (17.65)	
Ipsilateral MRND + contralateral SND	6 (6)		34 (24.46)		26 (34.21)		16 (47.06)	
Bilateral MRND	0 (0)		8 (5.76)		7 (9.21)		5 (14.71)	

Correlation analysis was performed using chi-square test for categorical variables and spearman’s rank correlation test for continuous/ordinal variables. A *p* value < 0.05 was considered statistically significant. Statistically significant differences are marked with an asterisk

*MRND* modified radical neck dissection, *SND* selective neck dissection

**Table 4:** Correlation of inconspicuous, accentuated (< 10 mm), enlarged (≥ 10 mm), and melted lymph nodes in contrast-enhanced computer tomography with histopathological characteristics of oral squamous cell carcinoma patients

Characteristics	Inconspicuous (%)	*p* value	Accentuated (%)	*p* value	Enlarged (%)	*p* value	Melted (%)	*p* value
No. of patients	100 (41.84)		139 (58.16)		76 (31.38)		34 (24.46)	
No. of resected LNs		0.570		0.570		0.299		0.027*
Median	32		39		43		49	
Range	9–121		10–110		13–110		14–110	
No. of metastatic LNs		< 0.001*		< 0.001*		< 0.001*		< 0.001*
Median	0		0		1		3	
Range	0–2		0–23		0–23		0–23	
Pathological T stage		< 0.001*		< 0.001*		< 0.001*		< 0.001*
T1	58 (58)		32 (23.02)		11 (14.47)		3 (8.82)	
T2	24 (24)		35 (25.18)		20 (26.32)		8 (23.53)	
T3	6 (6)		27 (19.42)		18 (23.68)		11 (32.35)	
T4	12 (12)		44 (31.65)		27 (35.53)		12 (35.29)	
Pathological N stage		< 0.001*		< 0.001*		< 0.001*		< 0.001*
N0	87 (87)		72 (51.80)		23 (30.26)		3 (8.82)	
N1	10 (10)		21 (15.11)		14 (18.42)		5 (14.71)	
N2a	1 (1)		4 (2.88)		3 (3.95)		1 (2.9)	
N2b	2 (2)		16 (11.52)		12 (15.79)		7 (20.59)	
N2c	0 (0)		5 (3.60)		5 (6.58)		3 (8.57)	
N3b	0 (0)		21 (15.11)		19 (25.00)		15 (42.86)	
Histological grading		< 0.001*		0.002*		< 0.001*		< 0.001*
G1	13 (13)		15 (10.79)		5 (6.58)		1 (2.94)	
G2	63 (63		55 (39.57)		26 (34.21)		9 (26.47)	
G3	23 (23)		66 (47.48)		43 (56.58)		22 (64.71)	
Gx	1 (1)		3 (2.16)		2 (2.63)		2 (5.88)	
Extranodal extension (% of LNMs)		0.091		0.119		0.006*		0.004*
ENE(−)	11 (84.62)		41 (61.19)		27 (50.94)		13 (41.94)	
ENE(+)	2 (15.38)		26 (34.21)		26 (49.06)		18 (58.06)	
Lymphovascular invasion		0.021*		0.018*		< 0.001*		< 0.001*
L0	98 (98)		126 (90.65)		65 (85.53)		26 (76.47)	
L1	2 (2)		13 (9.35)		11 (14.47)		8 (23.53)	
Vascular invasion		0.139		0.133		0.188		0.08
V0	100 (100)		136 (97.84)		74 (97.37)		32 (94.12)	
V1	0 (0)		3 (2.16)		2 (2.63)		2 (5.88)	
Perineural invasion		0.007*		0.005*		0.005*		0.003*
Pn0	92 (92)		110 (79.14)		57 (75.00)		23 (67.65)	
Pn1	8 (8)		29 (20.86)		19 (25.00)		11 (32.35)	

Correlation analysis was performed using chi-square test for categorical variables and spearman’s rank correlation test for continuous/ordinal variables. A *p* value < 0.05 was considered statistically significant. Statistically significant differences are marked with an asterisk

*ENE* extranodal extension, *LN* lymph node, *LNM* lymph node metastasis, *MRND* modified radical neck dissection, *SND* selective neck dissection

### Clinicopathological characteristics of patients with suspicious lymph nodes in computed tomography

Next, an analysis was conducted to examine the clinicopathological characteristics of patients with suspicious LNs in preoperative CT. Suspicious LNs observed in CT scans exhibited correlations with the type of ND, the number of metastatic LNs, pathological T stage, histopathological grading, lymphovascular invasion, and perineural invasion (all *p* < 0.05). Tumor localization demonstrated a correlation with accentuated LNs (*p* = 0.048), but not with enlarged or melted LNs (*p* > 0.05). Conversely, enlarged and melted LNs were associated with extranodal extension (*p* < 0.05). Additionally, melted LNs exhibited a correlation with the number of resected LNs (*p* = 0.027).

The gender distribution was fairly even across all groups. Furthermore, no significant age differences were observed (*p* > 0.05). Patients with enlarged and/or melted LNs tended to have tumors predominantly located at the floor of the mouth (26.32% and 35.29%), while those with accentuated LNs predominantly had tumors at the lateral tongue (26.62%).

Patients exclusively presenting accentuated cervical LNs were found to have T1 and T2 tumors in nearly 50% of cases (48.20%), whereas those with enlarged or melted LNs mostly had T2 and T3 tumors (50% and 55.88%). Particularly in the melted LNs group, the proportion of T3 tumors was the highest (32.35% vs. accentuated LNs group: 19.42% vs. enlarged LNs group: 23.68%). Interestingly, there was no disparity in the frequency of T4 tumors among the three groups (accentuated LNs group: 31.65% vs. enlarged LNs group: 35.53% vs. melted LNS group: 35.92%).

The percentage of pN0 and thus the false positive rate was the highest in the accentuated LNs group (51.80%), followed by the enlarged LNs group (30.26%), and was lowest in the melted LNs group (8.82%). Notably, pN3b status was found in 15.11% of the cases in the accentuated LNs group, in 25.00% of the patients in the enlarged LNs group, and in 42.86% of the patients in the melted LNs group.

Regarding tumor grading, well-differentiated tumors were observed in 10.79% of the patients in the accentuated LNs group, in 6.58% of the patients in the enlarged LNs group, and in 2.94% of the patients in the melted LNs group. Conversely, poorly differentiated tumors were more prevalent, with 47.48% in the accentuated LNs group, 56.58% in the enlarged LNs group, and 64.71% in the melted LNs group.

LNMs with extranodal extension were identified in 34.21%, 49.06%, and 58.06% of the patients, respectively. Additionally, lymphovascular invasion was found in 9.35%, 14.47%, and 23.53% of the patients, respectively. In contrast, tumor perineural invasion was found in 20.86%, 25.00%, and 32.35% of the patients, respectively.

Detailed clinicopathological characteristics based on LN appearance in CT scan and results of correlation analysis are presented in Tables 3 and 4.

### Diagnostic accuracy of contrast-enhanced computed tomography

Next, we examined the diagnostic accuracy of contrast-enhanced CT depending on the different LN presentations. Accentuated LNs demonstrated a sensitivity of 83.54% (95% confidence interval (CI) 73.14–90.61), specificity of 55.15% (95% CI 47.23–62.83), PPV of 47.14% (95% CI 38.72–55.73), and NPV of 87.50% (95% CI 79.22–92.91). Conversely, enlarged LNs exhibited lower sensitivity of 65.82% (95% CI 54.20–75.89; *p* > 0.05), but significantly higher specificity (84.84%; 95% CI 78.25–89.77; *p* < 0.05). Additionally, the PPV was significantly elevated in the enlarged LNs group (67.53%; 95% CI 55.79–77.51; *p* < 0.05). Notably, the NPV remained comparable between the two groups (accentuated LNs: 87.50%; 95% CI 79.22–92.91 vs. enlarged LNs: 83.83%; 95% CI 77.17–88.90; *p* > 0.05).

**Fig. 3:**
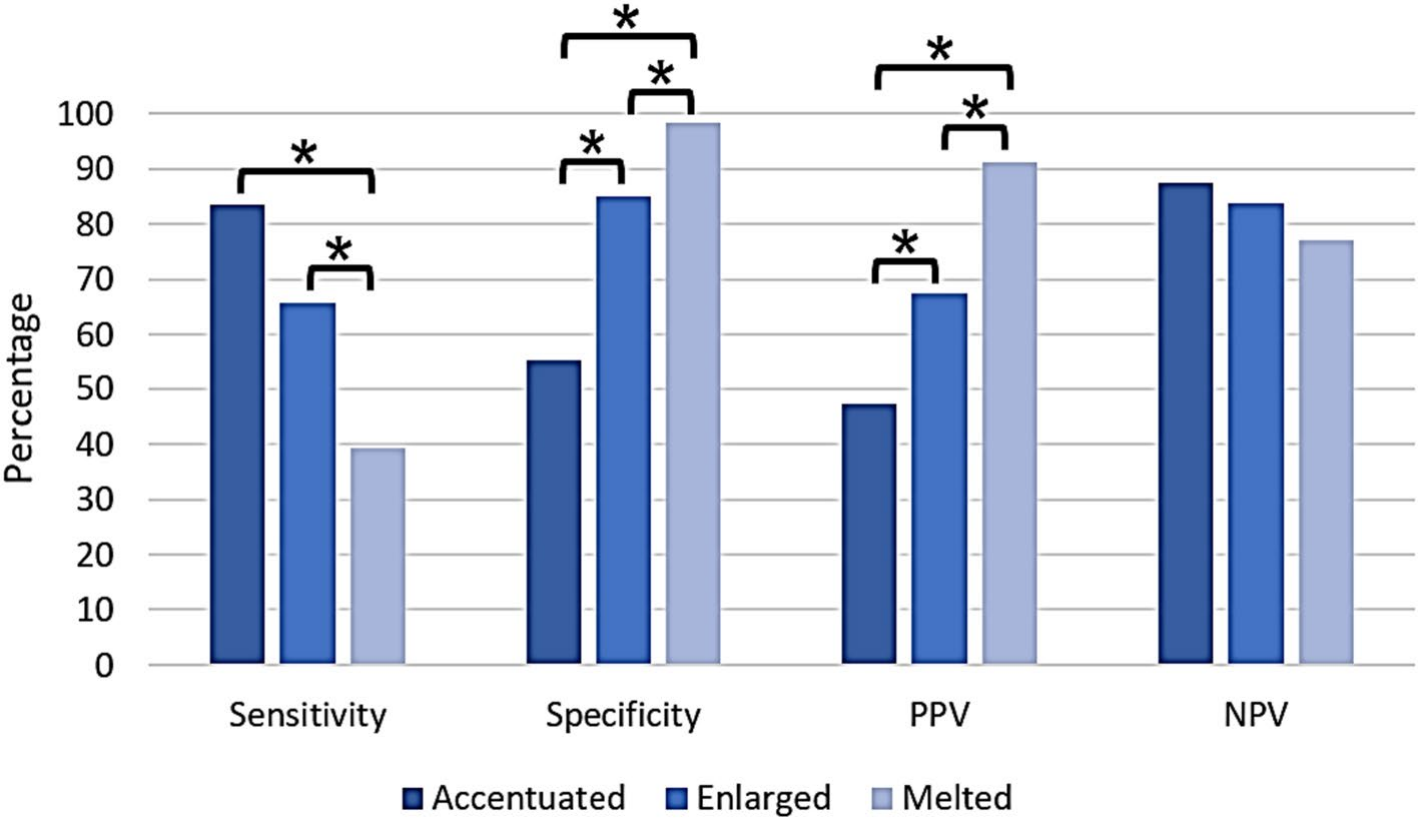
Diagnostic accuracy of contrast-enhanced computed tomography in anticipating lymph node status in patients with oral squamous cell carcinoma. Suspicious LNs were categorized into three groups: accentuated (< 10 mm), enlarged (≥ 10 mm), and melted. Sensitivity, specificity, positive predictive value (PPV), and negative predictive value (NPV) were compared across these groups. A t-test was employed for statistical analysis, with a significance level of *p* < 0.05. Statistically significant distinctions are denoted with an asterisk

Sensitivity was notably lower in the melted LNs group in comparison to both the accentuated and enlarged LNs groups (accentuated LNs: 83.54%; 95% CI 73.14–90.61 vs. enlarged LNs: 65.82%; 95% CI 54.20–75.89 vs. melted LNs: 39.24%; 95% CI 28.64–50.90; *p* < 0.05). In contrast, specificity and PPV demonstrated significant elevation in the melted LNs group (specificity: accentuated LNs: 55.15; 95% CI 47.23–62.83 vs. enlarged LNs: 84.84%; 95% CI 78.25–89.77 vs. melted LNs: 98.19%; 95% CI 94.36–99.53; PPV: accentuated LNs: 47.14%; 95% CI 38.72–55.73 vs. enlarged LNs: 67.53%; 95% CI 55.79–77.51 vs. melted LNs: 91.18%; 95% CI 78.19–97.69; all *p* < 0.05). Conversely, there was no notable distinction in NPV between the melted LNs group and the others groups (accentuated LNs: 87.50%; 95% CI 79.22–92.91 vs. enlarged LNs: 83.83%; 95% CI 77.17–88.90 vs. melted LNs: 77.14%; 95% CI 70.75–82.52; *p* > 0.05). Results of this statistical analysis are presented in Fig. 3 and Table 5.

**Table 5:** Sensitivity, specificity, positive and negative predictive value of contrast-enhanced computed-tomography in detecting lymph node metastases in oral squamous cell carcinoma depending on the image-based appearance

Characteristics of the LNs	Sensitivity (%)	Specificity (%)	PPV (%)	NPV (%)
Accentuated	83.54 (73.14–90.61)	55.15 (47.23–62.83)	47.14 (38.72–55.73)	87.50 (79.22–92.91)
Enlarged	65.82 (54.20–75.89)	84.84 (78.25–89.77)	67.53 (55.79–77.51)	83.83 (77.17–88.90)
Melted	39.24 (28.64–50.90)	98.19 (94.36–99.53)	91.18 (78.19–97.69)	77.14 (70.75–82.52)

*LN* lymph node, *PPV* positive predictive value, *NPV* negative predictive value

### Subset analysis: diagnostic accuracy of contrast-enhanced computed tomography depending on patients’ characteristics

Subsequently, we performed a subset analysis in order to delve deeper into the impact of different clinicopathological characteristics on the accuracy of CT. In general, accentuated LN demonstrated higher sensitivity compared to enlarged and melted LNs, although the differences were not universally significant. Significant sensitivity differences emerged between accentuated and melted tumors localized at the lower jaw, in female patients, poorly differentiated tumors, and T3/T4 tumors (*p* < 0.05), irrespective of age. However, no significant differences were noted between tumors localized at the upper jaw/buccal plane or in male patients (*p* > 0.05). Moreover, a significant sensitivity difference was observed between enlarged and melted LNs in T3/T4 tumors (*p* < 0.05).

The distinction between accentuated and enlarged LNs was significant in patients with T3/T4 tumors (*p* < 0.005), while it missed significance in other groups (*p* > 0.05).

The specificity of enlarged and melted LNs, regardless of characteristics, was generally higher than that of accentuated LN. Except for patients aged ≥ 65 years, there was a significant difference in specificity between accentuated and melted LNs in all patients (*p* < 0.05). In patients with tumors localized at the lower jaw, aged ≥ 65 years, male gender, with well- to moderately differentiated tumors, and T1/T2 tumors, specificity was significantly higher in melted LNs than enlarged LNs (*p* < 0.05). Additionally, a significant specificity difference was observed in female patients and those aged ≥ 65 years, independent of localization, T stage, and grading (*p* < 0.05). However, no significant difference was seen in male patients and patients under 65 years (*p* > 0.05).

PPV increased from accentuated LNs to melted LNs. A significant difference between accentuated and enlarged LNs was noted in tumors localized at the lower jaw (*p* < 0.05). Notably, there was a significant difference between enlarged and melted LNs in male patients and G1/G2 tumors, regardless of pathological T stages and age classification (*p* < 0.05).

Within our study population, no significant differences were identified concerning NPV (*p* > 0.05).

Table S2 and Fig. S1 provide an overview of sensitivity, specificity, PPV, and NPV based on sex, age, tumor localization, T stage, and grading.

### Comparison of diagnostic accuracy of contrast-enhanced computed tomography between subgroups

Subsequently, we compared sensitivity, specificity, PPV, and NPV between patient subgroups. The PPV of accentuated and enlarged LNs in contrast-enhanced CT was significantly higher in the group with poorly differentiated tumors (G3) compared to well- or moderately differentiated tumors (G1/G2; *p* < 0.05). Likewise, NPV was notably higher in the melted LNs group when investigating CTs of patients with poorly differentiated tumors (*p* < 0.05). This relationship also held for T3/T4 tumors, with a significantly higher PPV in the accentuated and enlarged LNs groups and a significantly higher NPV in the melted LNs group (*p* < 0.05). No significant differences in specificity and sensitivity were observed across all subgroups (*p* > 0.05). Figure S2 provides an overview of the comparison of PPV and NPV based on sex, age, tumor localization, T stage, and grading.

## Discussion

Accurately predicting the LN status preoperatively holds pivotal significance in determining the extent of ND for patients with OSCC. The implications of accurately managing LNs extend to not only affecting patient mortality (Wushou et al. 2021) and morbidity (Teymoortash et al. 2010), but also influencing their overall quality of life (Inoue et al. 2006). The progression from occult LNMs to clinically evident LNMs is linked to a compromised oncological outcome (Cai et al. 2020). Conversely, an overestimation of the preoperative stage could result in excessive treatment, escalating costs (Acevedo et al. 2016), and augmenting patient morbidity (Teymoortash et al. 2010).

The aim of this was study was to evaluate the diagnostic precision of contrast-enhanced CT in the detection of LNMs and to identify clinicopathological characteristics associated with its diagnostic accuracy.

In the realm of local staging of OSCC, contrast-enhanced CT and MRI serve as the established standards. Both modalities are endorsed by the current German staging guideline for assessing the primary tumor and evaluating cervical regions for LNMs. Typically, these image-based techniques are complemented by clinical examination and palpation (DGMKG 2021).

MRI is recognized for its superiority in measuring tumor thickness and local infiltration, whereas it tends to overestimate lymph node status and exhibit a higher false negative rate compared to CT (Goel et al. 2016; Lwin et al. 2012). MRI offers the advantage of superior soft-tissue contrast, enabling finer details in soft tissue recognition, and boasts fewer metal artifacts (such as those caused by dental fillings or implants). On the contrary, CT excels in evaluating cortical erosion (Tshering Vogel and Thoeny 2016).

When contemplating diagnostic options, cost and time efficiency must be taken into account. Opting for a single modality is more favorable than employing multiple options. MRI is associated with higher costs and time consumption compared to CT. In contrast, CT has downsides including metallic artifacts, radiation exposure, and the need to inject iodinated contrast medium, which carries the risk of contrast-induced nephropathy (Tshering Vogel and Thoeny 2016).

In Germany, CT tends to be the preferred choice due to its widespread availability, relatively low cost, efficiency, ease of use, objectivity, and reproducibility.

In this study, suspicious LNs identified through contrast-enhanced CT were categorized into three groups: accentuated (< 10 mm), enlarged (≥ 10 mm), and melted. In addition, patients who exhibited no clinical evidence of LNMs were differentiated.

In our patient cohort, cN0 status was verified in 87% of the patients. Remarkably, 13% of the patients exhibited LNMs despite the absence of suspicious LNs in CT, resulting in understaging.

In comparison to the study by Stoeckli et al., our false negative rate is low. Stoeckli et al. observed similar rates (approximately 24% in all groups) of understaged patients using methods such as ultrasound-guided fine-needle biopsy (FNB), 18F-fluorodeoxyglucose-positron-emission tomography/computed tomography (FDG-PET/CT), CT, and sonography (Stoeckli et al. 2012). In contrast, Pandeshwar et al. found a false negative rate of 8.6% in a group of 50 patients (Pandeshwar et al. 2013).

Overall, the suspicious LNs observed in CT were associated with various factors, including the type of ND, the number of metastatic LNs, pathological T stage, histopathological grading, lymphovascular invasion, and perineural invasion (all *p* < 0.05). Tumor localization demonstrated a correlation with accentuated LNs (*p* = 0.048), while no such correlation was found with enlarged or melted LNs (*p* > 0.05). On the contrary, both enlarged and melted LNs were linked to extranodal extension (*p* < 0.05). Melted LNs showed a correlation with the number of resected LNs (*p* = 0.027).

Across all groups, there was a balanced distribution of sex, and no significant age-related differences were observed. Patients with enlarged and/or melted LNs tended to have tumors more frequently localized at the floor of the mouth, whereas patients with accentuated LNs often had tumors situated at the lateral tongue. Patients with solely accentuated cervical LNs were more likely to present with smaller, well-differentiated tumors. However, when considering suspicious LNs in CT in general, there was a substantial risk (at least 57.48%) of encountering poorly differentiated tumors. Among the preoperative LN statuses, accentuated LNs exhibited the highest degree of overestimation (false positive rate, 51.80%), while melted LNs yielded the most reliable assessment (8.82%; accentuated LNs: 30.26%). Patients with melted LNs faced a 42.85% risk of having a pN3b status.

Furthermore, the likelihood of extranodal extension among LNMs was 34.21% in the group with accentuated LNs, 49.06% in the group with enlarged LNs, and 58.06% in the group with melted LNs.

Next, our study aimed to evaluate the reliability of contrast-enhanced CT in detecting LNM based on the cervical levels defined by Robbins. The assessment revealed that LNMs in level IIa and IIb were displayed in CT the most reliable, with 88.46% and 92.86% being accentuated, 76.92% and 78.57% enlarged, and 53.85% and 57.14% melted, respectively. In contrast, level III exhibited the least reliable display of LNMs in CT, where only 62.96% were enlarged and merely 44.44% were melted. This aspect should be taken into account when assessing CT scans in the future.

Some authors suggest that B mode sonography could be a comparable or even superior preoperative diagnostic tool for LNMs in head and neck squamous cell carcinoma. Sumi et al. found that B mode sonography outperformed CT in all levels except level II, where CT performed equally well. This observation was attributed to the larger size of LNs in level II on CT images (Sumi et al. 2001). However, it is essential to note the requirement of experienced clinicians and the high interobserver variability in sonography. In addition, it is important to acknowledge that additional imaging methods are necessary to ascertain the local extent of the tumor since sonography has limitations in staging primary tumors within the oral cavity’s bony conditions (Marchi et al. 2019). Moreover, sonography access is limited to the upper neck regions using a linear transducer (Hohlweg-Majert et al. 2009).

Next, we estimated sensitivity, specificity, PPV, and NPV in order to determine diagnostic accuracy of contrast-enhanced CT in anticipating LN status in OSCC patients.

In the present study, accentuated LNs exhibited a sensitivity of 83.54%, a specificity of 55.15%, a PPV of 47.14%, and a NPV of 87.50%. In contrast, enlarged LNs displayed a lower sensitivity of 65.82% but a significantly higher specificity of 84.84% (*p* > 0.05). Furthermore, the PPV was significantly elevated in case of enlarged LNs (67.53%, *p* < 0.05). The NPV was comparable between both groups (accentuated LNs: 87.50% vs. enlarged LNs: 83.83%, *p* > 0.05). Notably, sensitivity was significantly lower in the group of melted LNs in comparison to the accentuated and enlarged LNs groups (accentuated LNs: 83.54% vs. enlarged LNs: 65.82% vs. melted LNs: 39.24%, all *p* < 0.05). Conversely, the specificity and PPV were significantly higher in the melted LNs group (specificity: accentuated LNs: 55.15% vs. enlarged LNs: 84.84% vs. melted LNs: 98.19%; PPV: accentuated LNs: 47.14% vs. enlarged LNs: 67.53% vs. melted LNs: 91.18%; all *p* < 0.05) than in the other groups. However, no significant difference was observed regarding the NPV between the melted LNs group and the other groups (accentuated LNs: 87.50% vs. enlarged LNs: 83.83% vs. melted LNs: 77.14%, *p* > 0.05).

Our results parallel those of Stoeckli et al., who reported a sensitivity of 86.9%, specificity of 53.8%, PPV of 89.8%, and NPV of 46.7% (Stoeckli et al. 2012). Laimer et al. compared various techniques (B mode sonography, CT, MRI, FDG-PET) and reported a sensitivity of 95.00%, specificity of 63.90%, PPV of 59.4%, and NPV of 95.8% for CT. They utilized a cutoff value of 10 to 15 mm to designate a LN as metastatic. In their study, CT achieved the highest sensitivity (Laimer et al. 2020). Meanwhile, Nguyen et al. described a sensitivity of 81%, specificity of 88%, PPV of 86%, and NPV of 83%. Their criteria for cervical LNMs included size > 10 mm, contrast enhancement, central nodal necrosis, extracapsular spread, and overall cervical LN appearance (Nguyen et al. 2014). Pandeshwar et al. defined radiological criteria for LNMs as size ≥ 10 mm and the presence of central nodal necrosis, yielding a sensitivity of 92.0%, specificity of 84.0%, PPV of 85.1%, and NPV of 91.3% (Pandeshwar et al. 2013).

Nevertheless, it is important to highlight that a significant portion of the existing data is over a decade old (Nguyen et al. 2014; Pandeshwar et al. 2013; Stoeckli et al. 2012), thereby reducing its validity these days. This is attributed to the considerable advancements in the spatial resolution of CT scans, which have substantially enhanced the ability to detect small LNMs.

We performed a subset analysis to delve deeper into the impact of different clinicopathological characteristics on the accuracy of CT. The specificity of enlarged and melted LNs was consistently higher than that of accentuated LN, irrespective of the characteristics considered. However, there was no statistically significant difference observed between these groups for patients under the age of 65 (*p* > 0.05). Furthermore, no significant disparity in specificity emerged between enlarged and melted LNs for tumors localized at the upper jaw/buccal plane, G3 tumors, and T3/T4 tumors (*p* > 0.05).

Nonetheless, it is worth noting that all image-based diagnostic methods present results that significantly diverge from histopathological findings. The primary challenge in preoperative diagnosis lies in the inability of these methods, as well as clinical examination, to reliably detect a specific type of metastasis known as occult metastases, which affect around 25% of OSCC patients. LNMs below a certain size (e.g., micrometastases, defined as ≤ 2 mm) are consistently challenging to assess due to the limitations posed by the slice thickness in image-based techniques.

The decision to perform prophylactic ND hinges on the calculated risk of occult metastases as described previously. Naturally, this proportion is heavily reliant on the sensitivity of the employed pretreatment diagnostic methods. Some cancer centers have turned to FDG-PET/CT scans to improve nodal involvement predictions in OSCC patients. While FDG-PET/CT aids in diagnosing unknown primary tumors (cancer of unknown primary, CUP) or assessing glucose metabolism in neoadjuvantly treated tumors (Nissan et al. 2021), it still lacks the suitability for determining the extent of ND in OSCC patients due to its low sensitivity and frequent false positive findings (Stoeckli et al. 2012; Stuckensen et al. 2000), particularly for LNs smaller than 10 mm (Yamazaki et al. 2008).

Despite this, SND is associated with significant morbidity, prompting the exploration of less invasive alternatives such as sentinel lymph node biopsy (SNB) for treatment deintensification. Furthermore, interventions like ultrasound-guided FNB have been pursued to characterize uncertain cases.

SNB offers the advantage of revealing lymphatic drainage patterns while minimizing surgical morbidity (Alvarez Amezaga et al. 2007). However, it is not applicable for tumors located at the floor of the mouth (de Bree et al. 2021). Nevertheless, within a treatment regimen incorporating SNB, accurate preoperative image-based staging remains pivotal, as patients classified as cN0 undergo SNB while those with clinically evident LNMs or advanced tumor stages (T3/T4) undergo ND. In addition, histopathological examination of the SNB specimen guides subsequent intervention decisions, potentially leading to a supplementary complementary ND.

Yet, the body of data on SNB remains limited, particularly regarding prospective studies. A major drawback in most previous SNB studies has been the varying histologic scrutiny applied to non-sentinel LNs. However, at present, the recommendation regarding SNB should be limited to its application in patients with multiple comorbidities, providing the potential for decreased surgery duration and postoperative morbidity.

As for FNB, limited studies have examined its sensitivity in assessing LN status. While its efficacy is modest for cN0 necks (Borgemeester et al. 2008) and hence for detecting occult metastases (Chaturvedi et al. 2015), it proves valuable in verifying LNMs in palpable LNs before surgery (Gençoğlu et al. 2003; Takes et al. 1998). In the case of palpable LNs, ultrasound-guided FNB demonstrates higher specificity than CT (Gençoğlu et al. 2003), although overall diagnostic reliability remains comparable (Takes et al. 1998). Nevertheless, sensitivity is somewhat hindered by sampling errors and insufficient aspirated material for cytology (de Bree et al. 2021). Therefore, alternative strategies such as frozen section technique and an immediate histopathological examination during surgery should also be considered, provided that histopathological diagnosis of a LNM has immediate consequences on the subsequent surgical procedure.

To improve the diagnostic accuracy of CT, researches have explored the utilization of artificial intelligence (AI) along with deep learning models for predicting LNMs in oncologic patients. Ariji et al. el conducted a study evaluating the performance of deep learning in diagnosing LNMs in OSCC patients, and their findings were comparable to those of experienced physicians (Ariji et al. 2019). However, in cancer types other than OSCC, e.g. pancreatic adenocarcinoma, AI demonstrated superior performance compared to physicians (Bian et al. 2023). In the future, the potential integration of AI and deep learning holds promise for enhancing the effectiveness of LNMs detection in OSCC patients.

## Limitations of the study

Our study has certain limitations that warrant acknowledgment. To begin, its retrospective and single-center design introduces inherent biases. Nevertheless, it is noteworthy that our study boasts a sample size of 239 patients and a very homogenous patient cohort, distinguishing it from comparable studies with smaller study cohorts. Nonetheless, it is imperative to conduct large-scale prospective trials to substantiate the outcomes of our study.

In addition to the selection of the imaging modality, the criteria employed for categorizing a LN as metastatic remain somewhat ambiguous. The absence of globally standardized and widely accepted guidelines for defining malignancy in LNs complicates the interpretation of radiological observations. While size is the predominant criterion, other investigations incorporate additional factors such as central necrosis and contrast enhancement [30]. Nonetheless, our findings underscore the significance of considering size (≥ 10 mm) and the presence of central necrosis.

## Conclusion

Overall, contrast-enhanced CT imaging proves sufficient for anticipating LNMs in OSCC patients. However, up to now, no preoperative diagnostic modality can completely replace the histopathological assessment and the subsequent need for ND. Further exploration is especially required to determine whether other methods may be suitable or even confer advantages in particular cases.

Looking ahead, the potential integration of AI holds promise for enhancing the diagnostic accuracy of computed tomography in anticipating LN status. However, this prospect necessitates thorough exploration and investigation.

## Supplementary Information

Below is the link to the electronic supplementary material.

Supplementary file1 (DOCX 2351 KB)

## Data Availability

The data that support the findings of this study are available from the corresponding author upon reasonable request.

## Abbreviations

AI: Artificial intelligence
CI: Confidence interval
cN0: Clinically node negative
CT: Computed tomography
END: Extended neck dissection
FDG-PET/CT: ^18^F-fluorodeoxyglucose-positron-emission tomography/computed tomography
LN: Lymph node
LNM: Lymph node metastasis
MRI: Magnetic resonance imaging
MRND: Modified radical neck dissection
NPV: Negative predictive value
OSCC: Oral squamous cell carcinoma
PPV: Positive predictive value
SNB: Sentinel lymph node biopsy
SND: Selective neck dissection
