# Supplementary material for: Diagnostic accuracy of contrast-enhanced computed tomography in assessing cervical lymph node status in patients with oral squamous cell carcinoma
Source: J Cancer Res Clin Oncol. 2023 Oct 25;149(19):17437–50. doi: 10.1007/s00432-023-05470-y (PMC10657302; doi:10.1007/s00432-023-05470-y)
Supplement: Supplementary file 1 — Supplementary file1 (DOCX 2351 KB) [file 432_2023_5470_MOESM1_ESM.docx]

**
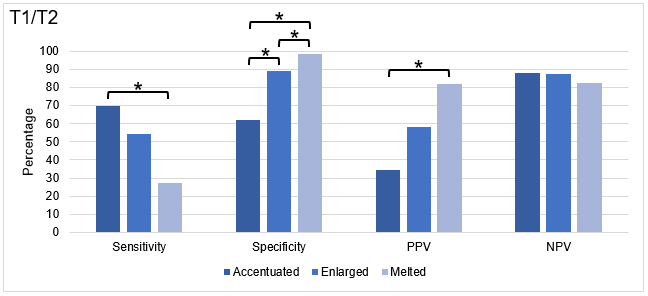

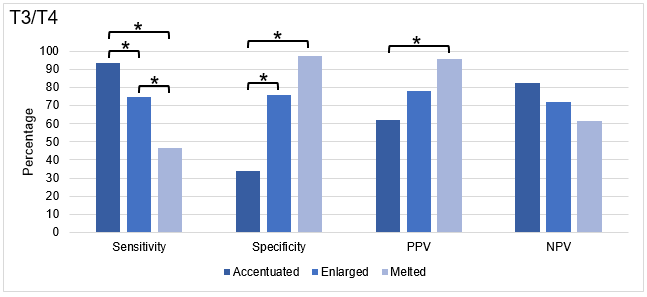
Supplementary information:** Diagnostic accuracy of contrast-enhanced computed tomography in assessing cervical lymph node status in patients with oral squamous cell carcinoma

Ann-Kristin Struckmeier MD, DMD, Ebrahim Yekta, Abbas Agaimy MD, Markus Kopp MD, Mayte Buchbender DMD, Tobias Moest MD, DMD, Rainer Lutz MD, DMD, Marco Kesting MD, DMD


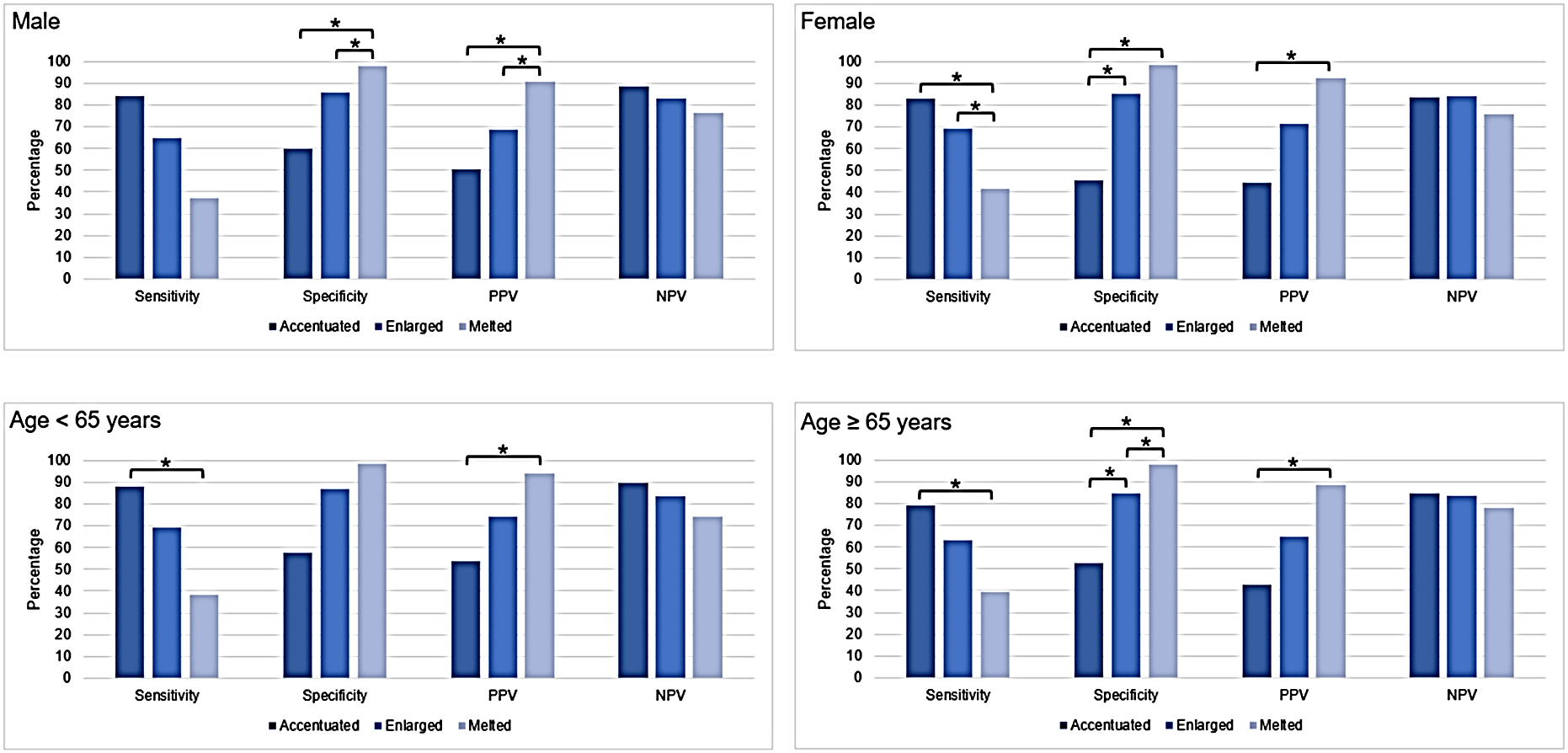


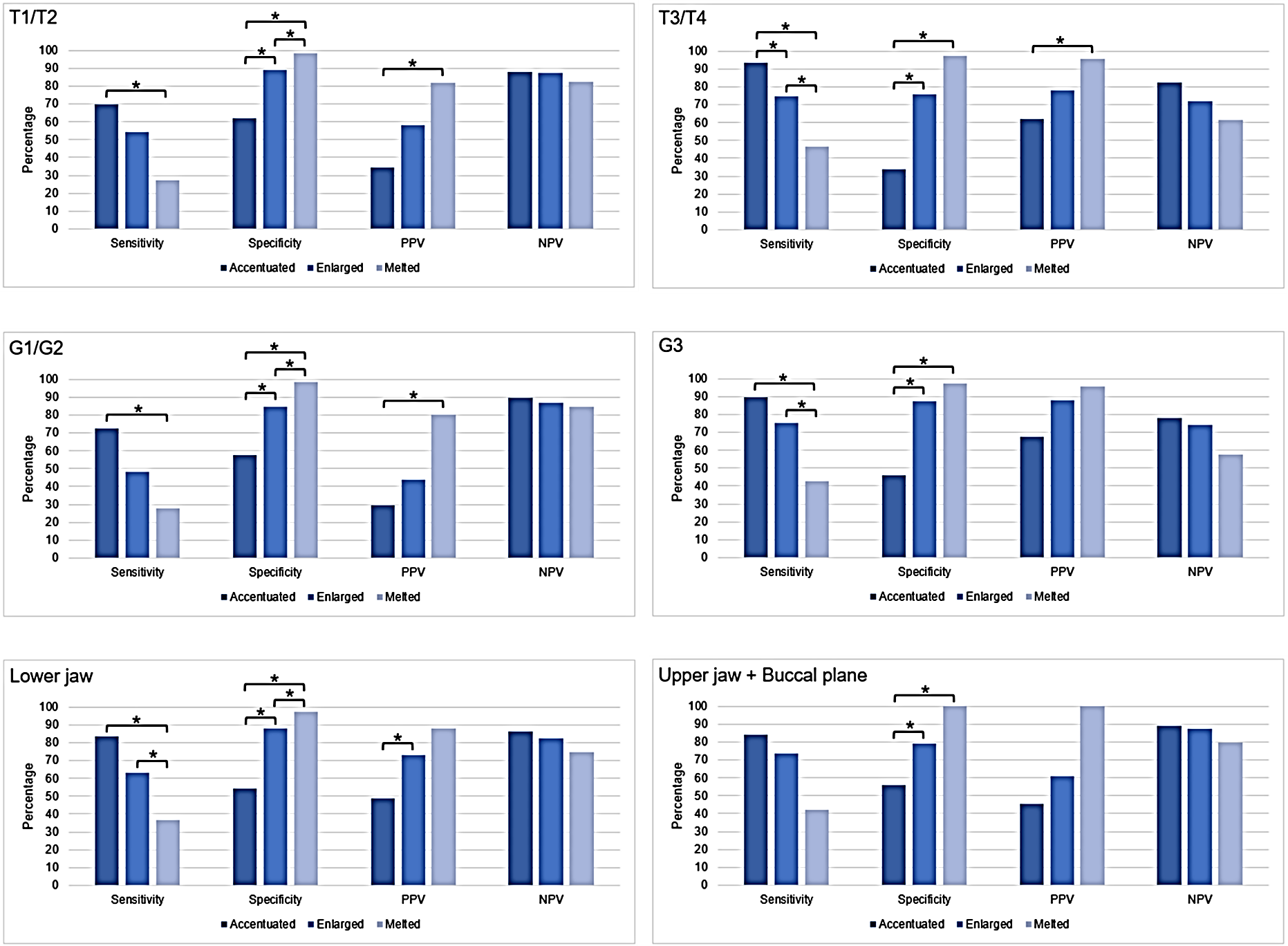


**Figure S1.** Comparative analysis of sensitivity, specificity, positive predictive value (PPV), and negative predictive value (NPV) of computed tomography in detecting lymph node metastases depending on sex, age, tumor localization, T stage, and grading. Suspicious LNs were categorized into three groups: accentuated (< 10 mm), enlarged (≥ 10 mm), and melted. A t-test was employed for statistical analysis, with a significance level of p < 0.05. Statistically significant distinctions are denoted with an asterisk.


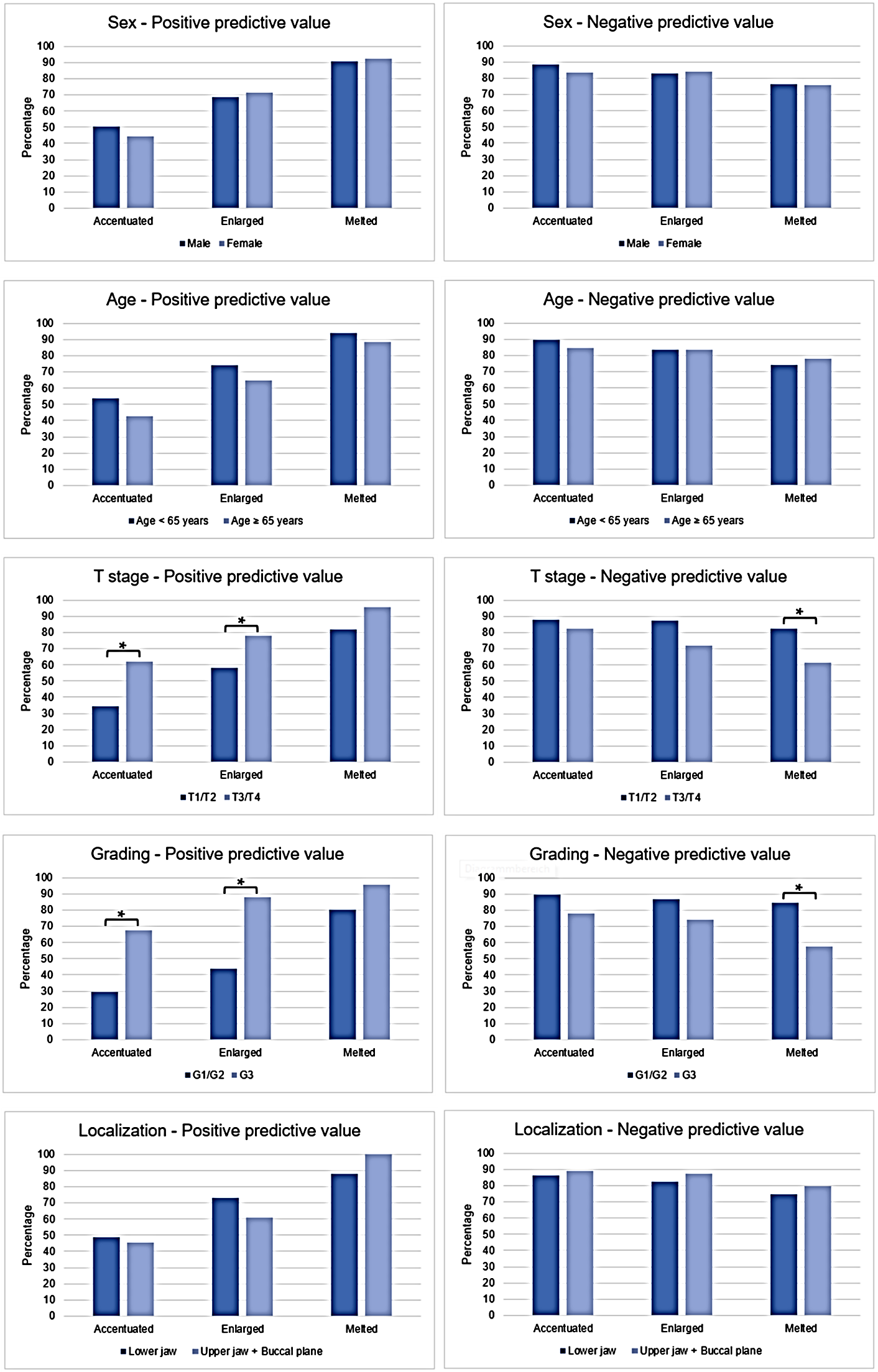


**Figure S2.** Comparative analysis of positive and negative predictive value between both sex, age < 65 years and ≥ 65 years, small (T1/T2) and advanced tumors (T3/T4), well-/moderately differentiated tumors and poorly differentiated tumors as well as tumors localized at the lower vs. at the upper jaw/buccal plane. Lymph nodes were categorized into three groups: accentuated (< 10 mm), enlarged (≥ 10 mm), and melted. A t-test was employed for statistical analysis, with a significance level of p < 0.05. Statistically significant distinctions are denoted with an asterisk.

**Table S1:** Inclusion and exclusion criteria

| **Inclusion criteria** | **Exclusion criteria** |
| --- | --- |
| - Time point of diagnosis between October 1, 2017, and October 31, 2022 - Primary oral squamous cell carcinoma - Preoperative staging with contrast-enhanced thin-section axial multidetector computed tomography with a minimal slice thickness of 1 mm - Primary surgical treatment including radical tumor resection and neck dissection - Neck dissection compromising at least the ipsilateral levels I-III and conducted as split up neck dissection | - Recurrent oral squamous cell carcinoma - Squamous cell carcinoma of the lip - No neck dissection conducted or with a decreased extent due to severe comorbidities |

**Table S2**. Sensitivity, specificity, positive and negative predictive value in detecting lymph node metastases in oral squamous cell carcinoma depending on the appearance of the lymph nodes in contrast-enhanced computed-tomography and clinicopathological characteristics

1. Localization: Lower jaw

| **Characteristics of the LNs** | **Sensitivity (%)** | **Specificity (%)** | **PPV (%)** | **NPV (%)** |
| --- | --- | --- | --- | --- |
| **Accentuated** | 83.33  (71.02-91.30) | 54.31  (44.83-63.50) | 48.54  (38.65-58.54) | 86.30  (75.79-92.88) |
| **Enlarged** | 63.33  (49.85-75.11) | 87.93  (80.26-93.00) | 73.08  (58.73-84.00) | 82.26  (74.15-88.31) |
| **Melted** | 36.67  (24.89-49.15) | 97.41  (93.06-99.33) | 88.00  (67.66-96.85) | 74.83  (67.00-81.38) |

1. Localization: Upper jaw + Buccal plane

| **Characteristics of the LNs** | **Sensitivity (%)** | **Specificity (%)** | **PPV (%)** | **NPV (%)** |
| --- | --- | --- | --- | --- |
| **Accentuated** | 84.21  (59.51-95.83) | 55.81  (40.01-70.59) | 45.71  (29.22-63.13) | 88.89  (69.70-97.09) |
| **Enlarged** | 73.68  (48.58-89.88) | 79.07  (63.52-89.42) | 60.87  (38.78-79.53) | 87.18  (71.77-95.18) |
| **Melted** | 42.11  (21.12-66.03) | 100  (89.79-100) | 100  (59.77-100) | 79.63  (66.08-88.93) |

1. Sex: Male

| **Characteristics of the LNs** | **Sensitivity (%)** | **Specificity (%)** | **PPV (%)** | **NPV (%)** |
| --- | --- | --- | --- | --- |
| **Accentuated** | 84.31  (70.86-92.52) | 59.62  (49.53-68.99) | 50.59  (39.60-61.52) | 88.57  (78.18-94.59) |
| **Enlarged** | 64.71  (50.00-77.20) | 85.58  (77.00-91.44) | 68.75  (53.60-80.91) | 83.18  (74.45-89.46) |
| **Melted** | 37.25  (24.47-51.95) | 98.08  (92.55-99.67) | 90.48  (68.17-98.33) | 76.12  (67.84-82.87) |

1. Sex: Female

| **Characteristics of the LNs** | **Sensitivity (%)** | **Specificity (%)** | **PPV (%)** | **NPV (%)** |
| --- | --- | --- | --- | --- |
| **Accentuated** | 82.76  (63.51-93.47) | 45.45  (32.19 -59.34) | 44.44  (31.16-58.51) | 83.33  (64.55-93.70) |
| **Enlarged** | 68.97  (49.05-84.02) | 85.45  (72.78-93.07) | 71.43  (51.13-86.05) | 83.93  (71.17-91.95) |
| **Melted** | 41.38  (24.09-60.87) | 98.18  (89.01-99.91) | 92.31  (62.09-99.60) | 76.06  (64.20-85.05) |

1. Age: < 65 J

| **Characteristics of the LNs** | **Sensitivity (%)** | **Specificity (%)** | **PPV (%)** | **NPV (%)** |
| --- | --- | --- | --- | --- |
| **Accentuated** | 88.10  (73.57-95.53) | 57.33  (45.40-95.53) | 53.62  (41.28-65.56) | 89.58  (76.56-96.10) |
| **Enlarged** | 69.05  (52.76-81.89) | 86.67  (76.39-93.08) | 74.36  (57.56-86.40) | 83.33  (72.82-90.49) |
| **Melted** | 38.10  (24.00-54.35) | 98.67  (91.79-99.93) | 94.12  (69.24-99.69) | 74.00  (64.10-82.03) |

1. Age: ≥ 65J

| **Characteristics of the LNs** | **Sensitivity (%)** | **Specificity (%)** | **PPV (%)** | **NPV (%)** |
| --- | --- | --- | --- | --- |
| **Accentuated** | 78.95  (62.22-89.86) | 52.38  (41.26-63.28) | 42.86  (31.28-55.22) | 84.61  (71.37-92.66) |
| **Enlarged** | 63.16  (46.00-77.71) | 84.52  (74.62-91.18) | 64.86  (47.42-79.28) | 83.53  (73.57-90.38) |
| **Melted** | 39.47  (24.49-56.55) | 97.62  (92.86-99.59) | 88.24  (62.25-97.94) | 78.10  (68.75-85.38) |

1. Grading: G1/G2

| **Characteristics of the LNs** | **Sensitivity (%)** | **Specificity (%)** | **PPV (%)** | **NPV (%)** |
| --- | --- | --- | --- | --- |
| **Accentuated** | 72.41  (52.51-86.55) | 57.63  (48.19-66.56) | 29.58  (19.63-41.75) | 89.47  (79.78-95.02) |
| **Enlarged** | 48.28  (29.89-67.10) | 84.74  (76.69-90.47) | 43.75  (26.84-62.12) | 86.96  (79.09-92.27) |
| **Melted** | 27.59  (13.45-47.49) | 98.31  (93.40-99.71) | 80.00  (44.22-96.46) | 84.67  (77.29-90.04) |

1. Grading: G3

| **Characteristics of the LNs** | **Sensitivity (%)** | **Specificity (%)** | **PPV (%)** | **NPV (%)** |
| --- | --- | --- | --- | --- |
| **Accentuated** | 89.79  (76.99-96.18) | 46.15  (30.43-62.62) | 67.69  (54.82-78.46) | 78.26  (55.79-91.71) |
| **Enlarged** | 75.51  (60.82-86.19) | 87.18  (71.77-95.18) | 88.10  (73.57-95.53) | 73.91  (58.59-85.25) |
| **Melted** | 42.86  (29.12-57.71) | 97.43  (84.92-99.87) | 95.45  (75.12-99.76) | 57.58  (44.82-69.44) |

1. Pathological T stage: T1/T2

| **Characteristics of the LNs** | **Sensitivity (%)** | **Specificity (%)** | **PPV (%)** | **NPV (%)** |
| --- | --- | --- | --- | --- |
| **Accentuated** | 69.70  (51.13-83.79) | 62.07  (52.55-70.78) | 34.33  (23.44-47.03) | 87.80  (78.27-93.68) |
| **Enlarged** | 54.55  (36.60-71.47) | 88.79  (81.26-93.66) | 58.06  (29.26-74.93) | 87.29  (79.59-92.47) |
| **Melted** | 27.27  (13.94-45.79) | 98.28  (93.89-99.70) | 81.82  (47.76-96.79) | 82.61  (75.02-88.33) |

1. Pathological T stage: T3/T4

| **Characteristics of the LNs** | **Sensitivity (%)** | **Specificity (%)** | **PPV (%)** | **NPV (%)** |
| --- | --- | --- | --- | --- |
| **Accentuated** | 93.61  (81.44-98.34) | 34.15  (20.56-50.67) | 61.97  (49.64-73.00) | 82.35  (55.80-95.33) |
| **Enlarged** | 74.47  (62.36-85.58) | 75.61  (59.36-87.09) | 77.78  (62.52-88.29) | 72.09  (56.10-84.17) |
| **Melted** | 46.81  (32.37-61.77) | 97.56  (85.59-99.87) | 95.6  (78.03-99.77) | 61.54  (48.62-73.09) |

**Abbreviation:** LN: lymph node.
